# Supplementary material for: Non-synaptic interactions between olfactory receptor neurons, a possible key feature of odor processing in flies
Source: PLoS Comput Biol. 2021 Dec 13;17(12):e1009583. doi: 10.1371/journal.pcbi.1009583 (PMC8668107; doi:10.1371/journal.pcbi.1009583)
Supplement: Attachment — Submitted filename: response_to_reviewers_ploscb.pdf [file pcbi.1009583.s019.pdf]

Response to Reviewers

We thank the reviewers for their thorough and constructive review of the manuscript. We have engaged with the review vigorously and have implemented most of the reviewer suggestions. Due to this, there are a lot of substantial changes to the manuscript. Apologies if the diff file looks a bit crowded.
Below we respond to the raised concerns point by point. Our responses are in **bold font**.

Reviewer #1

Major points

1. The authors show a detailed comparison between three models: control, LN model & NSI model. Each model implements a different computational mechanism in the olfactory pathway of the drosophila. This is indeed valuable for understanding how each one of those contributes to the hypotheses presented. However, the most interesting and realistic model, the one that considers both NSI and LN inhibition, is not explored. This “final” model is crucial for estimating the relations between all the interactions taking place and for comparing the results with real experimental data.
We followed the reviewer's suggestion and additionally tested a simple mixed model against the first two hypotheses. Briefly, results from this analysis do not change the main conclusions of this work, but reinforce it: NSIs can be helpful for encoding concentration ratio and concentration correlation. Indeed, the mixed model behaviour can be interpreted as a middle way between the LN-model and the NSI-model, even though for the case of correlation encoding we observed a partially synergistic effect. Please refer to each section of the first two hypotheses in the the Results (see pages 9 to 18), the final paragraphs in the Abstract and the Discussion (see page 20).
Through their detailed non-linear modeling work, the authors present interesting results about the influence that NSI has on various coding properties. However, the explanations the authors provide for the results are quite *descriptive*. It would be important to provide a quantitative simple phenomenological model with explanatory power, such that the most important factors underlying the effects presented will become more explicit. This would provide a much deeper understanding of the phenomena shown in the different hypotheses.
We agree with the reviewer that a quantitative description at an intermediate level between the “microscopic” level implemented by our quantitative model and the macroscopic level of the qualitative intuitions illustrated in figure 1 is desirable. However, such simplified phenomenological models are not always viable. We have introduced as much of this into the revised manuscript as was workable (see below).

For example, in Fig. 6, the most interesting panels are D (showing the effect of LNs on the R^{PN}) and E (showing the effect of NSIs to the R^{ORN}): what is the net inhibitory attenuation effect that is caused by the LNs to the input they receive? Is it a multiplicative effect? Can we divide panel D by panel C and get a constant? Is the effect more divisive/subtractive? What in the model is the most important factor for generating the effect of the NSIs? The fundamental components that control the PN output as well as how exactly it is done is not clear enough, and this can improve the paper phenomenologically.
Figure 4e illustrates intuitively the relationships between ORN and PN firing rates, i.e.

that it is sigmoidal. The effect is neither really multiplicative or additive - unfortunately, simple categorizations of this kind do not always apply. However, we included in the manuscript a figure illustrating the activity of PNs/ORNs for different values of the concentration and ratio to help the reader to link the results to the general intuition illustrated in figure 1b.

Same for Fig. 7: what is the single parameter that controls the effects presented?

We added a paragraph explaining more clearly that the effect is mainly dependent on the LN synaptic time scale. The information was already there, as supported by the comparative figure S9 but we made it more explicit now. The reason why NSIs do not show an interaction for asynchronous pulses is that NSIs are instantaneous while the LN inhibition has a finite time scale of the LNs and inhibitory synapses.

What is the principle that I need to hold in my working memory about the relationship between the ORNs and the PNs?

The main thing is the sigmoidal, and hence non-linear relationship illustrated in Fig. 4 which reproduces experimental results. We have fine-tuned the main text to reinforce this fundamental point.

2. I find two of the conclusions of the authors quite confusing and I suggest they would review them:

a. For the second hypothesis, the authors basically show negative result (in Fig. 7B, there is no effect for NSI on the output of PNs compared to the basic control condition).

Apologies if this wasn't clear in the original text. The observation that the NSI model does not show interference between the responses to two asynchronous odor pulses (original Fig. 7, now Fig. 8) is a positive result. In addition, NSIs can actually help encoding the correlation of the concentration and if we consider the high frequency such encoding is better than the one achieved by the LN model.

When the authors use a complex input scheme, they show that NSI may contribute to the readout of the temporal correlation between the two odors from one PN. However, this comes at the cost of less clear readout of each of the odors' concentration (because now the output of the PNs is not affected only by concentration but also by the correlation with the other odor).

Thank you for reinforcing this point. As we state throughout the manuscript, NSIs do disrupt the signal. What we are trying to argue is that this may have evolved to be tolerated because encoding the correlation in a plume could be more important than information about the absolute odour concentration. Keeping in mind how absolute concentration fluctuates unpredictably in odour plumes it may be a much less informational signal than the correlation structure of the fluctuations.

It is also important to say that correlation between odors can be read out in the control condition, except one needs PNs from both populations to do that. These issues should be discussed in the text (see also Fig 8 comment below).

The advantage of implementing this kind of signal elaboration at the periphery can be energetic or anticipatory.We have tried to now make this clearer in the Discussion section 'further hypotheses about NSIs'.

b. The conclusions of the third hypothesis are not clear. The authors should test if the effect of NSI remains when normalizing the curves to the maximum firing rate. In that case there could be actually a potential gain to NSI (depending on the results);

The dynamic range is calculated using certain percentages of the maximum firing rates of the ORNs. Therefore, a further normalization will not affect the result.

also, why did the authors look at the sum and not the difference/ratio between the dynamic range of the ORNs? It looks like at 10e-6 (in Fig. 10A) the difference between the curves is larger, which may help the next computational station, the PNs).

We selected the dynamic range and the detection threshold because they are important features commonly adopted to characterize the sensitivity of neurons and

because those quantities were analyzed by Vermeulen and Rospars 2004. We used the sum of the activity for similar reasons: 1. For simplicity, as the sum is the simplest way to represent the dynamics of both neurons together and 2. because it was resembling the measure adopted by Vermeulen et al. 2004 (both reasons are now explicitly stated in the manuscript). But we agree with the Reviewer on the arbitrariness of this choice and we hope that our study will spark the curiosity of other researchers interested in this field that will probably find other measures to test. Trying to help in this direction, we better described the changes generated by the NSIs on the dose-response curves in general and on the two fundamental thresholds (high and low) that determine the dynamic range in particular (see Fig. 11).

Additionally, the third hypothesis is ORN-specific, discussing how the dynamic range of the ORNs is affected by NSI. To complete the picture, this result should be quantified not only at the level of ORNs, but also with respect to their outputs, showing the implications of this result on the PNs. How does this effect change the dynamic range of the PNs and their encoding ability?

We are not sure we understand the point raised by the reviewer. For the first two hypotheses, a comparison with LN inhibition was essential because this mechanism has been proposed to fulfill the same tasks. However, for the dynamic range, no higher brain areas can change the ORNs dynamic range or their detection threshold. Therefore, for this hypothesis, if the dynamic range of ORNs is decreased, this will filter through directly to the dynamic range of PN responses and cannot be "undone" through processes in the AL. At best one could see that the decrease in dynamic range might be slightly less if the PNs themselves further decrease the overall dynamic range of the ORNs.

I also do not agree with the discussion of the nonmonotonic relationship in figure 10, which is negligible. Is the peak before the plateau in Fig 10A even statistically significant? If so, it should also be shown more explicitly with an inset in the figures.

In an ill-advised attempt to avoid cherry picking, we had reported a figure that was not properly illustrating the non-monotonic behavior. We have now added two additional figures that clearly illustrate the effect.

Given the current state of the results of hypothesis 3, the authors should look more deeply into it and keep the option to exclude it in the revision if the results do not justify it.

We believe that after following the reviewers' suggestions the results for this section of the paper are now sufficiently clear to form a valid and valuable addition to the paper.

Minor

<u>More details about 3b:</u>

In this context, figure 10 should be better explained:

- Lower values of the "ORNs similarity" represent higher similarity (log1=0), if I understand correctly, so it actually measures *dissimilarity*. The axis name should be changed.
  The reviewer is right. We corrected the x-axis label and the related part in the text.
- It should be better specified in the text that it is a "sensitivity similarity", as both respond to the stimulus in this analysis.
  corrected
- Line 404: *"First, the NSI mechanism decreases the lower threshold for ORNs that respond to the same odors":* This is true only for the dissimilar values. Also, please better explain in the text how you calculate the *combined* lower threshold value, and not only for one curve.
  we have now clarified this in the text
- "*Second, the NSI mechanism decreases the dynamic range for ORNs that respond to the same odor":* This is true only for similar ORNs.
  we have now clarified it in the text

- “*Third, both lower threshold and dynamic range are best when responses of paired ORNs are completely different, or, indeed, NSIs are absent”:* Why? Please better explain its merits and the reasons for this effect.
  We removed the final reference to absent NSIs, which admittedly is a bit confusing.
- “*Note that our control model is equivalent to the case where one ORN is not sensitive at all for the odorant”:* please explain. The dynamic range of the control curve (pink in B) is changed across the similarity axis, so it cannot be true. Do you mean the “single ORN” dashed black line?
  This attempt of relating presence or absence of NSIs to situations where the two neurons respond similarly or differently was ill-advised and confusing. We have removed this sentence.
- How do you explain the ctrl graph in 10b,c? Why would the ORN similarity affect the ctrl in such a way?

  It is indeed at first surprising that the dynamic range of the control model appears to depend on the neuron sensitivities even when they don’t interact. However, if two neurons have disparate sensitivities their dynamic ranges combine into a larger overall dynamic range. Intuitively, for the low end of the combined dynamic range the more sensitive neuron contributes more and for the high end, the less sensitive neuron provides more information. We have rewritten this section and redesigned the figure, including for the lower threshold, to be more clear in the manuscript.

<u>Text (line numbers):</u>

205: There are no LNs in the control model, so to state that they are not affected is confusing.
corrected

226: I see a sigmoidal relationship between ORNs and PNs in figure 4e. It does not explain why there is a distortion from concentration to ORNs as in fig. 4f we observe a linear relationship. This point should be better clarified.
In fig. 4f the x axis has a log-scale, while in 4e the x axis has a linear scale.

231: The reduction in the ceiling effect is clear for the PNs (Fig. 6d), not for the ORNs in 6c.
Panels c and d refer to the LN-model, therefore ORN activity for this model is not different from the control-model.

254: extra space.
corrected

275: unnecessary “dot and dashed line” – was stated in 273.
we removed it

319: rephrase this sentence, too complex. **Removed it and incorporated it in the previous paragraph.**

354: “Fig. 9a”.
corrected

378: “aspects”
corrected

456: “Fig. 1e”.
corrected

Figure-related comments:

Figure 1: This figure should be better designed:

- Present the hypotheses you explore more explicitly in the figure itself (not only in the legend). **corrected**
- Panel B, inset: it looks like the effect you are looking for is very small. **Apologies, if this was confusing but this figure is just a motivational illustration and not based on data.**
- Panel D: “No NSI” is confusing as the authors also have a model without NSI, but here the meaning is that NSIs are not involved. **corrected**
- E arrives after B. **The figure should be read column-wise.**
- Consistency: odor/odour, panel letters font size, font type, au/a.u., ORN rates/response, conc/conc. **corrected**
- Match all the colors in the figure: in panel A, the ORN color hues don’t match the green and purple in the rest of the figure. **corrected**

Fig 5: This figure should be better designed:

- The alignment between the model illustration in A and the waveforms in B makes one think that they match. I would try and illustrate the models in a small and more concise manner.
- It should be noted that only one of the ORNs is drawn even though both are stimulated.
- Panel B on the right: I don’t see a “dot and dashed line” and a “shaded area”. I guess that the “dot and dashed line” is on top of the “dashed line” – this should be better presented. If this is the case and the lines are one on top of each other, currently it looks like the message is “the effect on the PNs is the same for both the LN and NSI models”. This is not the case of course, as you show in the next figure. I would want to see this panel for different total concentrations (say 1 and 10), to grasp the concentration-dependent manner of the effect.
- B: The titles of the columns (“Input”, “ORN”, “PN”) should be moved down a bit. It looks like they are related to panel A.
- Consistency: panel letters font size
- A: the word “NSI” is cut.

We completely redesigned Fig.5 by separating the panels into two figures and we tackled all the issues indicated. For this figure, the results for a single pulse for NSI, LN and control are very similar and for LN and control are practically identical.

Fig 6:

- Fig 6e: what is the predicted experimental dynamic range? Could you deduce that from Su et al. (2012) or Zhang et al. (2019) and fit it to the data? **No, unfortunately the information from the experimental literature is still not sufficient for this aim.**
- G: I don’t see further explanation in “Model and methods”. Please add this. **See the previously accidentally omitted “Analysis” section in the Methods**
- Figure captions: You say that the legend is in F but it is in D. Also, there are six different overall concentrations, and not four, as described in the captions. **corrected**

Fig 7:

- A: Add legend.
  We added a legend.
- B: so, no advantage for the NSI over the ctrl model?
  We are studying a possible disadvantage here: The disruption of encoding of the intensity at zero correlation, or in other words, for asynchronous pulses. That the NSIs do not disrupt this is a positive effect. We have tried to make this more clear in the text.

Fig 8:

- Panels B, C: is the average firing rate only across neurons or is it only for a specific neuron across time? This should be better explained.
  We added that they are averaged over neurons and time.
  Why would a system use the average firing rate from a single neuron to deduce correlation if it can use the input from both PNs at different timings to get this?
  That is one of the main questions we tried to answer with this work and fig. 7 and fig. 8 are a partial answer. Another part of the answer resides in the efficiency (less spikes wasted) and time precision (NSIs takes place earlier and more locally at the level of the sensilla) for the NSI.
- “PN activity” should be more specific (same for Fig. 9).
  PN activity is defined as “the total firing rate above a given threshold”. We have tried to be more thorough and explicit in the main text.

Fig 10:

- A: What is the ORN similarity in this example?
  We changed this panel and added the ORN similarity.
- B,C: What is OM? Move legend to top left.
  OM stays for order of magnitude, we now added this in the caption.
- C: No caption for this panel.
  corrected

<u>Table 1:</u>

- $N_{ORN,PN}$ = 20? In Fig. 2 you specify 80.
  We corrected fig. 2
- Please verify all parameters here.
  We have verified them and corrected omissions and mistakes.

<u>Methods:</u>

- Better to add a description for C in equation (2) and V_K in equation (3).
  We added a description for C and V_K.

Reviewer #2

MODEL & METHODS

1) Missing descriptions in the “model and methods” section

Unfortunately, in the submitted MS version parts of the method section seem to be missing. The paper repeatedly refers to the methods section for detailed explanations but I could not find

any of the expected explanations. This makes it hard and often impossible to fully understand and review the MS.

Apologies for the missings and at times confusing/incorrect parts of the Methods. We carefully reviewed the whole MS and addressed all the points indicated by the reviewer.

For example:

- Line 240 mentions the coding error without clear definition (measured over time course? For peak responses?) and caption of Fig. 6 refers to the method section, but nothing is there. It is also mentioned as either squared relative distance or relative distance at different instances of the text body.
  The correct definition is as in your brackets. See the previously accidentally omitted "Analysis" section in the Methods
- how is firing rate estimated? **See "Analysis" section in the Methods**
- ratio of peak activity? **See "Analysis" section in the Methods**
- Line 307 "The statistics of the plumes and how we simulated them are described in detail in the Model and methods; …" – no, nothing there. A detailed description is particularly relevant if others would like to use this input model for comparison.
  See previously accidentally omitted "Simulation of realistic plumes" section in the Methods
- Line 603 "In addition to the inputs from ORNs, PNs also receive global excitation from PNs associated with other glomeruli and from other parts of the brain." I could not identify where and how this global excitation enters the model. The noise in equation (6) has zero mean and there is no other excitatory input specified. Does equation (6) lack a term for constant current input or similar?
  This was a general statement and should have said "which we do not model here". But as this is quite confusing, especially in the Methods section, we have now removed this sentence. There is no such other input into the PNs in our models.

2) Model parameters

- Table 1 – It would be helpful to indicate e.g. by bold font which of the parameters have been fitted to experimental data and which have not. By what method/approach was the fitting of all 24 parameters performed?
  We indicated the parameters not used for the fitting (with an asterisk) and the methods used for the fitting. We added the methods used for the different fittings (e.g. brute force to find good a starting point and to refine the fit a Trust Region Reflective hill climbing algorithm)
- The LIF ORN is defined as voltage equation and parameters are provided as dimensionless numbers except for resting, reversal and threshold voltages. This choice makes it difficult to interpret these parameters relative to the parameters of the other neurons. What is the dimension of the adaptation time constant? Please clarify.
  We have now rewritten the equation in the same form as for PNs and LNs and added the appropriate units of measure to the relevant parameters in Table 1 so all equations are presented in the same format
- Please carefully check nomenclature in 4.2. E.g. a_y, b_y, b_r versus alpha_y , beta_y, beta_r.
  Thanks, we corrected them.
- Which parameter is meant with d_r in line 621: "The parameters ay and by are estimated together with br and dr to reproduce the data presented in [28]." – does this refer to the

parameter alpha_r?
Yes, we corrected it.

- Capacity “C” for PN and LN is not given.
Sorry, now added, C= 1nF.
- Please carefully check for other parameters that might not be defined such as e.g. concentration.
We added some text about this that explains how the dimensionless value of c should be interpreted (see “Olfactory Receptor Neurons” section).

RESULTS

3) Concentration ratio

- Fig. 6f shows one of the central results that is very interesting. The ratios of response rates show a clear picture. What are the absolute rates, e.g. of the dominant odor? Are these still in a realistic range?
We added the values for the ORNs and PNs in the supplementary materials. Yes, the ORN and PN responses are still realistic, in particular the weaker ORNs decrease their activity and their firing rate therefore goes towards zero (as illustrated in Fig.1b).
- I do not understand Fig. 6g: “It is clear that NSI model outperforms both LN and control model, apart for very high concentrations.” In Fig. 6a-f the authors look at the concentration ratio of the two odorants without providing absolute concentrations for any of the two odours. What is the absolute concentration in a-f? In Fig. 6g. only an absolute concentration is provided on the y-axes for the 2d calibration plot. Which concentration ratio was chosen for panel g? This is puzzling, please clarify and use the caption to provide full information.
The reviewer is right: We were not perfectly clear on that figure. We have added a clarification in the text (see section ‘NSIs help encoding odorant concentration ratio’ and now Fig.7). In Fig.6g (now 7i) the y axis is the value of the absolute concentration for the odorant with lower concentration. Each colour value represents the overall similarity of the full curve of response ratios to the input ratio for a fixed concentration of the less concentrated odour, i.e. summarises the deviation of one of the lines in fig. 6a-f (now 7a-h) from the diagonal.

4) Correlation analysis

- The analysis in Fig. 8 is difficult to assess due to the missing explanation of the plume statistics. The caption reads “… response … to two 4 s long realistic plumes with statistical properties as described in the text;” but I cannot find this description.
We added the relevant description of the plumes simulation in the Methods section.
- For panel d it says that the max. whiff duration equals 3ms. This is on the very short end of the statistics, does this hold for all results in Fig. 8?
Thanks for indicating the typo: Instead of 3ms we should have written 3s.

5) Adaptation

- The chosen model of spike frequency adaptation in the ORNs introduces a mode of self-inhibition. Table 1 indicates an ORN adaptation time constant of 0.0035 - is this to be interpreted as 3.5ms – please clarify?
Sorry for the confusion, it should have read “decay rate of adaptation” and we should have indicated the units of kHz. This hence corresponds to 3.5 Hz or an adaptation decay timescale of roughly 286 ms.

How does this number fit to time constants reported in the experimental and recent model literature that investigated ORN adaptation (e.g. ref 28 and others not referenced)? A time constant of ~3ms would seem extremely short (e.g. compared to 258ms in the PN) and I

would expect to see no dynamic effect of adaptation in the resulting response profile because the adaptation current effectively decays over less than one ISI. However, the firing rate dynamics in Fig. 3 looks realistic to me, both in amplitude and temporal evolution. I would have expected that the phasic-tonic responses to a step stimulus are due to the adaptation. However, a decay time constant of 3.5ms and a maximum rate of 100Hz corresponding to an average 10ms inter-spike interval indicates that the adaptation profile of the firing rate (adapting over the course of several hundreds of ms) is not due to the spike frequency adaptation. Can the authors explain mechanistically how this response rate adaptation is brought about? If it is due to the ORN adaptation current then I would like to see the response in Fig. 3 when adaptation is switched off for comparison (possibly with compensating average negative charge effect).
The conundrum is resolved by the fact that the 286ms timescale we used is compatible with recent findings and comparable to the PN's. The visible adaptation in the ORN firing profile is caused by this adaptation current exactly as the reviewer expected.

- L 544 the authors argue that adaptation is an important element of odor processing. If the adaptation time constant in the ORN spike generator has really been set to only 3.5ms then I doubt that it is of relevance in the authors' ORN model.
As above, the timescale is much larger, sorry for the confusion.
- The assumingly short ORN adaptation time constant might have little effect on asynchronous mixture encoding. A longer realistic time constant of tens to hundreds of ms (similar to the very long LN inhibitory synaptic time constant) might have?
As above, the timescale is actually fairly long.

6) Lateral inhibition

- The synaptic time constant tau_LN is set to 250ms as a standard (compared to 19ms for tau_PN) and thus is of the same duration as the PN adaptation time constant (tau_ad=258ms). Is that a realistic number? To me synaptic time constants of 20ms seem already long, 250ms seems very specific. Where the synaptic time constants among the fitted parameters or were they chosen freely? Are there numbers in the literature that can support these long time constants or is this a model prediction? Since LNs are non-adapting, could the LN->PN inhibition have a similar effect as the PN adaptation, i.e. an outward conductance that can sum up over the stimulus period with similar long time constants as the PN adaptation?
[Otis, Koninck and Mody, J Physiol 463:391-407 (1993)] have measured time constants of $GABA_B$ synapses in rat brain slices and found "At 34-35 °C, the average activation time constant (τ_m) was 45.2 ms, while the two inactivation time constants (τ_{h_1} and τ_{h_2}) were 110.2 and 516.2 ms. It is not unreasonable to assume that slow lateral inhibition in the AL could be based on $GABA_b$ synapses, following e.g. the model of Bazhenov [Bazhenov, Stopfer, Rabinovich, Huerta, Abarbanel, Sejnowski, Laurent, Neuron 30(2): 553-567 (2001)]. In addition, we have already tested a shorter timescale of 25ms, which might be more realistic for $GABA_A$, see Figure S11 and S12.
- In line 303 the authors state that the NSI "… *distort responses less than LN inhibition in the case of asynchronous mixtures*." However, as they state this is not the case for 'short' inhibitory synaptic time constants of 25ms (which to me still seems a very long synaptic time constant). Thus, this seeming disadvantage of the LN model depends on the choice of time constant. This needs to be made explicit here and in the Discussion.
The effect is reduced at tau_LN=25ms, but it is still present. We now carefully describe these relationships in the manuscript.

7) PN response saturation

- The result in Fig. 4E is nice and fits well the experimental observations. Which mechanism causes the saturation effect in the PN firing rate with respect to ORN input rate? The authors should explain the causes of this effect.
  As reported before (Kim et al., Lazar et al.,), the PNs activity can be approximated by a linear sum of the ORNs response and the first derivative of the ORNs response. The adaptation is the mechanism that yields this effect (at least in our model). One consequence of this is the saturation effect in the PN firing rates. However, this result could probably be further improved once a model with all the receptor types is implemented; with that model we could test several additional hypotheses proposed by Olsen and Wilson 2010.

8) Above threshold rate encoding

- The authors state that "A biologically plausible and commonly adopted hypothesis argues that information is conveyed in the high activity, i.e. firing rates above a given threshold." The authors are asked to provide references here. I find this approach rather critical. Results in Fig. 8 and supplements seem to depend critically on this chosen threshold. Given the avg. PN rates of 30 – 45 Hz in Fig. 8c and multiplying those by 5 PNs the total activity would be around 150 – 225 Hz. The 150Hz threshold criterion thus implies that most of the PN activity as base activity is neglected.
  As described in the MS, our perspective on this result is the following: the PN average activity is probably not really interesting as it takes into account all the information (or lack of) coming from the baseline response even during zero concentration periods (blanks). A very simple and easy to implement measure of meaningful responses is the average firing rate above a given minimum response threshold. This measure is based on the biologically plausible hypothesis that information is conveyed in the high activity, i.e. firing rates above a given threshold, while activity below the threshold is simply baseline noise activity. We did not indicate a specific reference demonstrating that a high frequency based measure is biologically plausible, as most of the studies in Cognitive Neuroscience root their decision making models on the same principle: Above a given threshold of firing rates a decision is made. The average activity for the NSI model is around 40Hz (see present Fig. 9c), as was well observed by the Reviewer, and therefore for the three employed thresholds most of the PNs' activity is discarded. However, we don't think it is a problem to discard part of the signal that carries mainly noise. More importantly, the NSI model is at least equally good at encoding the correlation between odorant concentrations as the LN-model for low frequency threshold, and for very high frequency threshold (>150Hz) the NSI model outperforms the LN model. Given that the goal of the work is to analyse possible explanations for the existence of this interaction, we note that 1. Even if the NSI model performs similarly to the LN model, it could be advantageous to use it as it is more efficient than LN inhibition (less energy wasted in spikes), and 2. The NSI model outperforms the LN model in a determined regime (high frequency), again reinforcing its plausible utility.
  The figure below illustrates the distribution of firing rates during the response to a plume stimulus. There is a large peak of values below 50 Hz which corresponds to baseline firing and a second peak around 130 Hz which corresponds to the responses to whiffs. The measure of "peak activity" is motivated by this observation.

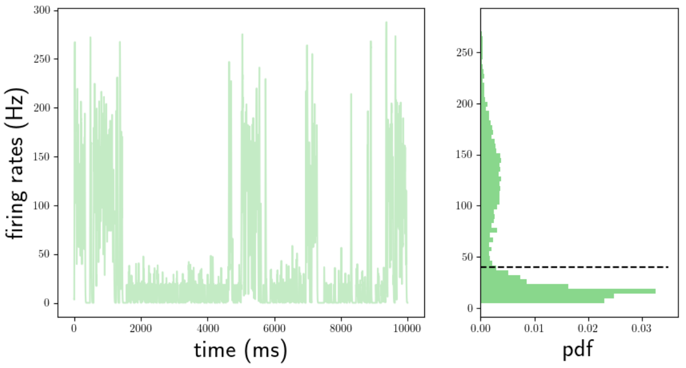	PNs activity and probability distribution for 50s simulation of real plumes with wmax=3s, rhos=3, rhocs-.66.

- What does “Total PN activity above 150 Hz.” (caption Fig. 8) actually mean? Total as the sum across all 5 PNs per glomerulus? What does a number of 4.000 on the y-axes of Fig. 8d mean? Is this an integrated measure as indicated by line 449 in the Discussion? Why are units of this measure arbitrary, should this be total number of spikes as in time-integrated rate or completely meaningless? Please specify the definition of this threshold model in the text and method section. Again, the method section does not describe this approach at all.
We apologize for the lack of clear information. We added a section in the Methods and clarified the related text in the Results. In particular, we added that: “for simplicity we used three measures for the activity of the PNs: the average, maximum and the peak activity. The average activity is measured as the average rate of the neurons of the same glomerulus over 200ms after the stimulus onset. The maximum activity is measured as the maximum rate of each neuron within 200 ms after stimulus onset, averaged across all neurons of the same glomerulus. For the longer, more realistic odorant plumes, we used the definition of peak activity as the integrated activity over time windows where the neuron firing rate is above a given threshold (e.g. 50, 100, 150 Hz).”
- In light of this unclear definition I find it hard to interpret line 452f “… this also adds further evidence in favor of using peak activity to encode important features of a signal, in this case stimulus correlations, as hypothesized in earlier work (see e.g. [19, 52]).” What exactly do the authors mean here with “peak activity” with respect to their analyses: the peak of a time-varying rate above some high threshold? The integrated rate above some threshold? And what definition of peak activity has been used in the two references? Are these definitions coherent?
We removed that sentence.

DISCUSSION

9) As the authors state, the model does not capture the underlying physics of the NSI mechanism (line 480 f). While implementing the underlying mechanism into the model is clearly out of the scope of this study, it would however be interesting to shortly review what is hypothesized to be relevant mechanisms behind NSIs and/or discuss plausible mechanisms.
The mechanism(s) underlying NSIs is a very interesting topic, but for now we do not have a theory for it beyond the already discussed work of Vermeulen et al. that would be worth the speculation.

10) A number of studies have shown that both, NSIs and LI, are key features in the olfactory processing in the fly. In the conclusion of the manuscript, the authors shortly mention that it would be interesting to study their differential roles when both mechanisms are present (line 593 f.). In my opinion, joint and mutual effects of NSIs and LI should be discussed in more detail. How do the two mechanisms possibly complement each other (e.g. narrow and specific connectivity between two ORNs in sensilla and broad connectivity/inhibition through LNs in AL)?
Even though knowing exactly how each of these components is relevant for the elaboration of the olfactory signal is clearly an important and interesting field of study, in our opinion, this discussion goes beyond the scope of this paper, that is focused on the question: why is a typically disruptive mechanism allowed to take place between ORNs? We have tried to give a partial answer with our computational study. It is partial because, for example, there could of course be a synergistic effect between LN and NSI inhibition. And it is partial because we only tackled three possible hypotheses with several assumptions (due to our limited amount of knowledge). So we built our study to respond to these questions: Are NSI helpful for the three given hypotheses and wouldn't it be easier to just use LN inhibition? We are convinced, as others before us, that these questions are interesting. Moreover we are happy that this work raises new curiosities and questions to answer.
However, nudged by the observations of both reviewers, we have now included what could be an interesting starting point for a future study: a proto-analysis of a mixed model where both inhibitory effects are present. We added for the first two hypotheses new results with a model combining both NSI and LN inhibition (see pages 9 to 18) and we changed the final paragraphs in the Abstract and in the Discussion (see page 20).
See also the answer to issue #1 of Reviewer #1.

MINOR

1. Fig.5: input in b, left panel is dimensionless? The standard deviation over 10 trials is not visible and essentially zero; thus I assume that the displayed rate is in each trial averaged across all 80 ORNs and 5 PNs in the right panel? How was firing rate estimated, this is not indicated in the methods?
The input to the ORN is for simplicity adopted as dimensionless. Yes, the average is over all neurons. We added the firing rate calculation description in Methods.
2. L 224: “Fig.6 based on the ratio of peak activity R = nu_b/nu_a (both for ORNs and PNs, see Model and methods) during the first 200 ms after the stimulus onset.” Unfortunately the methods section does NOT explain the stimulus protocol or response estimates of nu_a, nu_b. It would be helpful to make clear here that this is related to the peak firing rate responses and again the method for rate estimation should be defined in the method section.
See previously accidentally omitted section Analysis in the Methods.
3. Correlation analysis in Fig. 8: I understand that the correlation among odor inputs is simulated and controlled by some model parameter (model not described, see 1 above). Why does the x-axes then read “Observed correlation”? Is there any additional step of correlation analysis that leads to these x values? Please clarify.
See previously accidentally omitted section “Simulation of realistic plumes”. As correctly intuited and observed by the Reviewer, given the stochasticity of the simulated plumes their correlation is not constant and it depends on the parameters specified in the Methods section “Simulation of realistic plumes”.
4. L 355: w_max = 50m vs. 50s ?
corrected

5. L 372: The subscripts should read “NSI” or “LN” (and not “x”) in both subtraction terms, respectively.
   corrected
6. Fig. 5a is only mentioned in the text in line 195 ff. (“The whiffs in plumes (see e.g. Fig.S5a) are mimicked with simple triangular odorant concentration profiles that have a symmetric linear increase and decrease (see Fig. 5a).” I think this statement refers to Fig. 5b and apart from that, Fig. 5a is not mentioned in the text.
   corrected
7. Fig. 5b: The difference between the dashed NSI line and the dot-dashed LN line is not very clear (applies even more to PN panel in Fig. 7a). Here, color coding / more different line styles would increase discriminability.
   For Fig. 5: The three models have very similar and realistic responses and therefore they are not so distinguishable. For fig. 7: Only the LN line is separated from the others in the PN column, and it is clearly indicated in the caption. We increased the zoom for maximum visibility.
8. The Figure referencing in inconsistent (e.g. “see Fig.1b” in line 54; “see Fig.1, panels c-d” in line 56; “8a-b” in line 329)
   corrected
9. Some subfigures are referenced wrongly (line 197: Fig.5b instead of Fig.5a; caption of Figure 6: “two odorants for four different overall concentrations (colours, see legend in f)” should be “six different concentrations” and “see legend in d”)
   corrected
10. Caption for Fig 10c is missing
    corrected
11. Check line 355: “from 0.01 to 50 m” should be “0.03” and “s” ?
    corrected
