# Supplementary material for: Non-synaptic interactions between olfactory receptor neurons, a possible key feature of odor processing in flies
Source: PLoS Comput Biol. 2021 Dec 13;17(12):e1009583. doi: 10.1371/journal.pcbi.1009583 (PMC8668107; doi:10.1371/journal.pcbi.1009583)
Supplement: Attachment — Submitted filename: 2021, Pannunzi, Non-synaptic interactions between olfactory receptor neurons.docx [file pcbi.1009583.s018.docx]

Pannunzi and Nowotny examine the function of non-synaptic interactions (NSIs) in the olfactory system of drosophila, an intriguing phenomenon that has been gaining attention in recent years.

They explore the functional effect of the NSI phenomenon in different models that take into account combinations of the following components in the olfactory pathway:

- The olfactory receptor neurons in the sensillum (with/without NSIs between them).
- Lateral inhibitory neurons in the antennal lobe.
- Projection neurons.

The modeling work is solid and well-designed, constraining the parameters on biophysical data and considering ORN adaptation and the statistics of realistic plumes.

The work focuses on three important sensory encoding properties that the NSIs have an impact on:

- The encoding of the ratio of odorant concentrations.
- The processing of the temporal characteristics of odorants.
- The effect on the dynamic range of ORNs when both ORN are sensitive to the same stimulus.

The authors claim that the models they compared show an added value of NSIs for the first two properties, showing a clear advantage of the NSI mechanism over lateral inhibition in the antennal lobe. For the last property, they present a disadvantage for the encoding of odorants using the NSI mechanism. Overall, my view is that the work is solid and has good potential for publication, but there are three major aspects that deserve attention before publication can be recommended: (1) Adding another condition to the modeling. (2) Better quantitative explanation of the results. (3) Answers to alternative interpretation for some of the conclusions.

**Major points**

1. The authors show a detailed comparison between three models: control, LN model & NSI model. Each model implements a different computational mechanism in the olfactory pathway of the drosophila. This is indeed valuable for understanding how each one of those contributes to the hypotheses presented. However, the most interesting and realistic model, the one that considers both NSI and LN inhibition, is not explored. This “final” model is crucial for estimating the relations between all the interactions taking place and for comparing the results with real experimental data.
2. Through their detailed non-linear modeling work, the authors present interesting results about the influence that NSI has on various coding properties. However, the explanations the authors provide for the results are quite *descriptive*. It would be important to provide a quantitative simple phenomenological model with explanatory power, such that the most important factors underlying the effects presented will become more explicit. This would provide much deeper understanding of the phenomena shown in the different hypotheses. For example, in Fig. 6, the most interesting panels are D (showing the effect of LNs on the R^PN^) and E (showing the effect of NSIs to the R^ORN^): what is the net inhibitory attenuation effect that is caused by the LNs to the input they receive? Is it a multiplicative effect? Can we divide panel D by panel C and get a constant? Is the effect more divisive/subtractive? What in the model is the most important factor for generating the effect of the NSIs? The fundamental components that control the PN output as well as how exactly it is done is not clear enough, and this can improve the paper phenomenologically.

Same for Fig. 7: what is the single parameter that controls the effects presented? What is the principle that I need to hold in my working memory about the relationship between the ORNs and the PNs?

Additionally, to further strengthen the functional understanding of this system, this work could benefit from a dynamical analysis of the system to explore its temporal characteristics and the effect of perturbations/modulations to it, but I understand if this is not in the scope of the manuscript.

3. I find two of the conclusions of the authors quite confusing and I suggest they would review them:
   a. For the second hypothesis, the authors basically show negative result (in Fig. 7B, there is no effect for NSI on the output of PNs compared to the basic control condition). When the authors use a complex input scheme, they show that NSI may contribute to the readout of the temporal correlation between the two odors from one PN. However, this comes at the cost of less clear readout of each of the odors’ concentration (because now the output of the PNs is not affected only by concentration but also by the correlation with the other odor). It is also important to say that correlation between odors can be readout in the control condition, except one needs PNs from both populations to do that. These issues should be discussed in the text (see also Fig 8 comment below).
   b. The conclusions of the third hypothesis are not clear. The authors should test if the effect of NSI remains when normalizing the curves to the maximum firing rate. In that case there could be actually a potential gain to NSI (depending on the results; also, why did the authors look at the sum and not the difference/ratio between the dynamic range of the ORNs? It looks like at 10e-6 (in Fig. 10A) the difference between the curves is larger, which may help the next computational station, the PNs).

Additionally, the third hypothesis is ORN-specific, discussing how the dynamic range of the ORNs is affected by NSI. To complete the picture, this result should be quantified not only at the level of ORNs, but also with respect to their outputs, showing the implications of this result on the PNs. How does this effect change the dynamic range of the PNs and their encoding ability?

I also do not agree with the discussion of the nonmonotonic relationship in figure 10, which is negligible. Is the peak before the plateau in Fig 10A even statistically significant? If so, it should also be shown more explicitly with an inset in the figures.

Given the current state of the results of hypothesis 3, the authors should look more deeply into it and keep the option to exclude it in the revision if the results do not justify it.

**Minor**

More details about 3b:

In this context, figure 10 should be better explained:

- Lower values of the “ORNs similarity” represent higher similarity (log1=0), if I understand correctly, so it actually measures *dissimilarity*. The axis name should be changed.
- It should be better specified in the text that it is a “sensitivity similarity”, as both respond to the stimulus in this analysis.
- Line 404: *"First, the NSI mechanism decreases the lower threshold for ORNs that respond to the same odors”:* This is true only for the dissimilar values. Also, please better explain in the text how you calculate the *combined* lower threshold value, and not only for one curve.
- *“Second, the NSI mechanism decreases the dynamic range for ORNs that respond to the same odor”:* This is true only for similar ORNs.
- *“Third, both lower threshold and dynamic range are best when responses of paired ORNs are completely different, or, indeed, NSIs are absent”:* Why? Please better explain its merits and the reasons for this effect.
- *“Note that our control model is equivalent to the case where one ORN is not sensitive at all for the odorant”:* please explain. The dynamic range of the control curve (pink in B) is changed across the similarity axis, so it cannot be true. Do you mean the “single ORN” dashed black line?
- How do you explain the ctrl graph in 10b,c? Why would the ORN similarity affect the ctrl in such a way?

Text (line numbers):

205: There are no LNs in the control model, so to state that they are not affected is confusing.

226: I see a sigmoidal relationship between ORNs and PNs in figure 4e. It does not explain why there is a distortion from concentration to ORNs as in fig. 4f we observe a linear relationship. This point should be better clarified.

231: The reduction in the ceiling effect is clear for the PNs (Fig. 6d), not for the ORNs in 6c.

254: extra space.

275: unnecessary “dot and dashed line” – was stated in 273.

319: rephrase this sentence, too complex.

354: “Fig. 9a”.

378: “aspects”

456: “Fig. 1e”.

Figure-related comments:

Figure 1: This figure should be better designed:

- Present the hypotheses you explore more explicitly in the figure itself (not only in the legend).
- Panel B, inset: it looks like the effect you are looking for is very small.
- Panel D: “No NSI” is confusing as the authors also have a model without NSI, but here the meaning is that NSIs are not involved.
- E arrives after B.
- Consistency: odor/odour, panel letters font size, font type, au/a.u., ORN rates/response, conc/conc.
- Match all the colors in the figure: in panel A, the ORN color hues don’t match the green and purple in the rest of the figure.

Fig 5: This figure should be better designed:

- The alignment between the model illustration in A and the waveforms in B makes one think that they match. I would try and illustrate the models in a small and more concise manner.
- It should be noted that only one of the ORNs is drawn even though both are stimulated.
- Panel B on the right: I don’t see a “dot and dashed line” and a “shaded area”. I guess that the “dot and dashed line” is on top of the “dashed line” – this should be better presented. If this is the case and the lines are one on top of each other, currently it looks like the message is “the effect on the PNs is the same for both the LN and NSI models”. This is not the case of course, as you show in the next figure. I would want to see this panel for different total concentrations (say 1 and 10), to grasp the concentration-dependent manner of the effect.
- B: The titles of the columns (“Input”, “ORN”, “PN”) should be moved down a bit. It looks like they are related to panel A.
- Consistency: panel letters font size
- A: the word “NSI” is cut.

Fig 6:

- Fig 6e: what is the predicted experimental dynamic range? Could you deduce that from Su et al. (2012) or Zhang et al. (2019) and fit it to the data?
- G: I don’t see further explanation in “Model and methods”. Please add this.
- Figure captions: You say that the legend is in F but it is in D. Also, there are six different overall concentrations, and not four, as described in the captions.

Fig 7:

- A: Add legend.
- B: so, no advantage for the NSI over the ctrl model?

Fig 8:

- Panels B, C: is the average firing rate only across neurons or is it only for a specific neuron across time? This should be better explained. Why would a system use the average firing rate from a single neuron to deduce correlation if it can use the input from both PNs at different timings to get this?
- “PN activity” should be more specific (same for Fig. 9).

Fig 10:

- A: What is the ORN similarity in this example?
- B,C: What is OM? Move legend to top left.
- C: No caption for this panel.

Table 1:

- N_ORN,PN_ = 20? In Fig. 2 you specify 80.
- Please verify all parameters here.

Methods:

- Better to add a description for C in equation (2) and V_K in equation (3).
